# Earlier consolidation and improved knee function of medial open wedge high tibial osteotomy with autologous bone graft

**DOI:** 10.1007/s00590-023-03656-1

**Published:** 2023-08-04

**Authors:** Eva-Maria Bodenbeck, Jessica C. Böpple, Julian Doll, Franziska Bürkle, Gerhard Schmidmaier, Christian Fischer

**Affiliations:** 1 Center for Orthopaedics, Ultrasound Center, Trauma Surgery and Spinal Cord Injury, Trauma and Reconstructive Surgery, Heidelberg University Hospital, Schlierbacher Landstraße 200a, 69118 Heidelberg, Germany; 2 Shoulder and Elbow Surgery, Arcus Sports Clinic, 75179 Pforzheim, Germany

**Keywords:** Knee osteoarthritis, Open wedge high tibial osteotomy, Bone graft, Consolidation, CEUS, DCE-MRI

## Abstract

**Purpose:**

Medial knee osteoarthritis can be treated with medial open wedge high tibial osteotomy (OWHTO). We sought to investigate osseous consolidation of the osteotomy with and without autologous bone grafts (ABG) to detect possible benefits of ABG in osseous healing and functional outcome.

**Methods:**

In this prospective study, patients without graft transplantation were compared to those receiving ABG after medial OWHTO. They were followed up 6 weeks, 12 weeks, 6 months and 12 months postoperatively. Radiographic progress of consolidation, clinical scores, contrast-enhanced ultrasound (CEUS) and dynamic contrast-enhanced magnetic resonance imaging (DCE-MRI) were assessed at each appointment.

**Results:**

A total of 35 patients were enrolled, 20 without and 15 with graft transplantation. Radiologic evaluation showed a significantly earlier consolidation of the osteotomy gaps (*p* = 0.012) in patients with ABG, resulting in a significantly higher rate of consolidation 12 months after surgery (60% without bone graft vs. 100% with bone graft, *p* = 0.006). At 6 weeks as well as 6-month follow-up, a tendency of earlier consolidation with ABG was apparent, but not statistically significant (6 weeks: 50% vs. 80%, *p* = 0.089; 6 months: 30% vs. 60%, *p* = 0.097). CEUS and DCE-MRI showed physiological perfusion of the osteotomy gaps in both groups. A tendency to better function and less pain in patients with ABG was recognizable.

**Conclusion:**

In our study, autologous bone grafting evocated earlier osseous consolidation after medial OWHTO and showed a tendency to a better functional outcome.

## Introduction

Knee osteoarthritis affected 263 million people globally in 2017 [1]. In combination with genu varum deformities, the risk of knee osteoarthritis can even be aggravated [2]. The open wedge high tibial osteotomy (OWHTO) is a well-accepted treatment option for osteoarthritis with satisfying short- and long-term results in elderly as well as in young patients [3]. Advantages of OWHTO are the preservation of bone stock, an easy bone exposure and correction during surgery as there is no need to involve the fibula [4]. On the other hand, there are some possible and severe complications, e.g. loss of correction, delayed bone union or even non-union [4]. To potentially avoid these disadvantages, autologous bone grafts (ABG), allografts or synthetic bone grafts can be used to fill the osteotomy gap [5]. Previous studies reported better results of ABG compared to other graft types [5]. Nevertheless, bone grafting can potentially lead to pain and complications at the donor location e.g. infections, fractures and sensory disorders [4]. Therefore, grafting with ABG should be performed after careful considerations. Most studies could not prove an accelerated consolidation or better knee function by using a graft [4, 6, 7]. However, a prospective randomized study reported that using a graft expedites the radiographic healing of the osteotomy gap, but no advantages regarding knee function were found [8].

Contrast-enhanced ultrasound (CEUS) and dynamic magnetic resonance imaging (DCE-MRI) were used in previous trials to examine the perfusion of bone healing in fractures, non-unions and septic non-unions [9–12]. In this trial, these two modalities were integrated to control physiological consolidation beyond common x-rays and to secure the comparability of both groups as well as detecting risk factors like infections and impaired perfusion.

The aim of this prospective study was to evaluate the use of ABG in osteotomy gaps of medial OWHTO as well as the influence of grafting on the postoperative knee function.

## Materials and methods

### Patients

Between 2015 and 2018, a total of 41 patients with medial knee osteoarthritis undergoing medial OWHTO at our hospital were included. Inclusion criteria were a minimum age of 18 years, varus deformity with medial knee osteoarthritis and written informed consent.

Exclusion criteria were a follow-up period less than one year, clinical infection, contrast agent intolerance (SonoVue® [Bracco, Milan, Italy] or Dotarem® [Guerbet, Villepint, France]) as well as respiratory insufficiency and recent myocardial infarction.

All patients with planned OWHTO surgery were included, regardless of the severity of the varus alignment or risk factors for impaired consolidation.

This study was conducted in accordance with the current declaration of Helsinki and was approved by the local Ethics Committee (S-033/2014). All patients agreed with the study protocol and gave their written informed consent.

Out of 41 patients who met the inclusion criteria, six patients had to be excluded: Five were lost to follow-up and one was excluded because of a clinical infection (ABG group). The remaining 35 patients were divided into two groups after surgery regarding the use of graft transplantation: Twenty patients did not receive a graft (group A), whereas in fifteen patients autologous bone grafting was performed (group B). The decision of grafting was made by the surgeon intraoperatively considering the osteotomy gap size as well as existing risk factors for impaired bone healing. There were no other criteria regarding the use or origin of the grafts.

No significant differences between groups were seen in age (42 (IQR: 31–49) and 44 (IQR: 27–47) years in group A and B), sex (35% vs 47% female), BMI (27.2 (IQR: 21.7–31.4) and 28.9 (IQR: 23.8–31.4) kg/m^2^ in group A and B) or medical history. Surgeons decided to use ABG transplantation more often in smokers (Table 1).

**Table 1:** Table of demographic data of group A (without ABG) in comparison to group B (with ABG)

	Group A (without graft)	Group B (with graft)	*p*-value
*n*	20	15	
Age median (IQR) [years]	42 (31, 49)	44 (27, 47)	0.580
Gender			0.510
Female	7 (35%)	7 (47%)	
Male	13 (65%)	8 (53%)	
BMI	27.2	29.0	0.540
Median (IQR) [kg/m^2^]	(21.7, 31.4)	(23.8, 31.4)	
Smoker			0.27
✖	17 (85%)	7 (47%)	
** ✔**	3 (15%)	8 (53%)	
Heart disease			0.430
✖	20 (100%)	14 (93%)	
** ✔**	0 (0%)	1 (7%)	
Diabetes			1.000
✖	19 (95%)	15 (100%)	
** ✔**	1 (5%)	0 (0%)	
Kidney disease			1.000
✖	20 (100%)	15 (100%)	
** ✔**	0 (0%)	0 (0%)	
Liver disease			1.000
✖	20 (100%)	15 (100%)	
** ✔**	0 (0%)	0 (0%)	
Osteoporosis			0.180
✖	20 (100%)	13 (87%)	
** ✔**	0 (0%)	2 (13%)	
Number of diseases			0.950
1	6 (30%)	4 (27%)	
2	4 (20%)	2 (13%)	
3	5 (25%)	5 (33%)	
4	1 (5%)	2 (13%)	
5	2 (10%)	1 (7%)	
6	2 (10%)	1 (7%)	

*BMI* body mass index; *HKA difference* difference of the hip-knee-ankle angle before and after surgery; *IQR* interquartile range; *p* < 0.05 is considered as statistically significant

### Differences in surgical technique between groups

During medial OWHTO, group B received a gap filling with ABG from different sampling points including the ipsilateral proximal tibia or iliac crest. Both groups received the TomoFix® Medial High Tibial Plate (DePuy Synthes, Switzerland).

### Postoperative assessment

Postoperatively, after increasing weight-bearing stepwise, most patients reached full load after 4 to 6 weeks. Range of knee motion was not limited. Physiotherapy and lymph drainage 2 to 3 times a week were recommended.

Follow-up appointments were performed at 6 weeks, 12 weeks, 6 months and 12 months after surgery. At each appointment, patients were asked to answer questionnaires including the Knee Society Score (KSS), the Lysholm Knee Score (LKS) and the SF12 [13–15]. Radiographic evaluation as well as a physical examination was performed by an experienced senior orthopedic surgeon.

### Radiographic evaluation

**Fig. 1:**
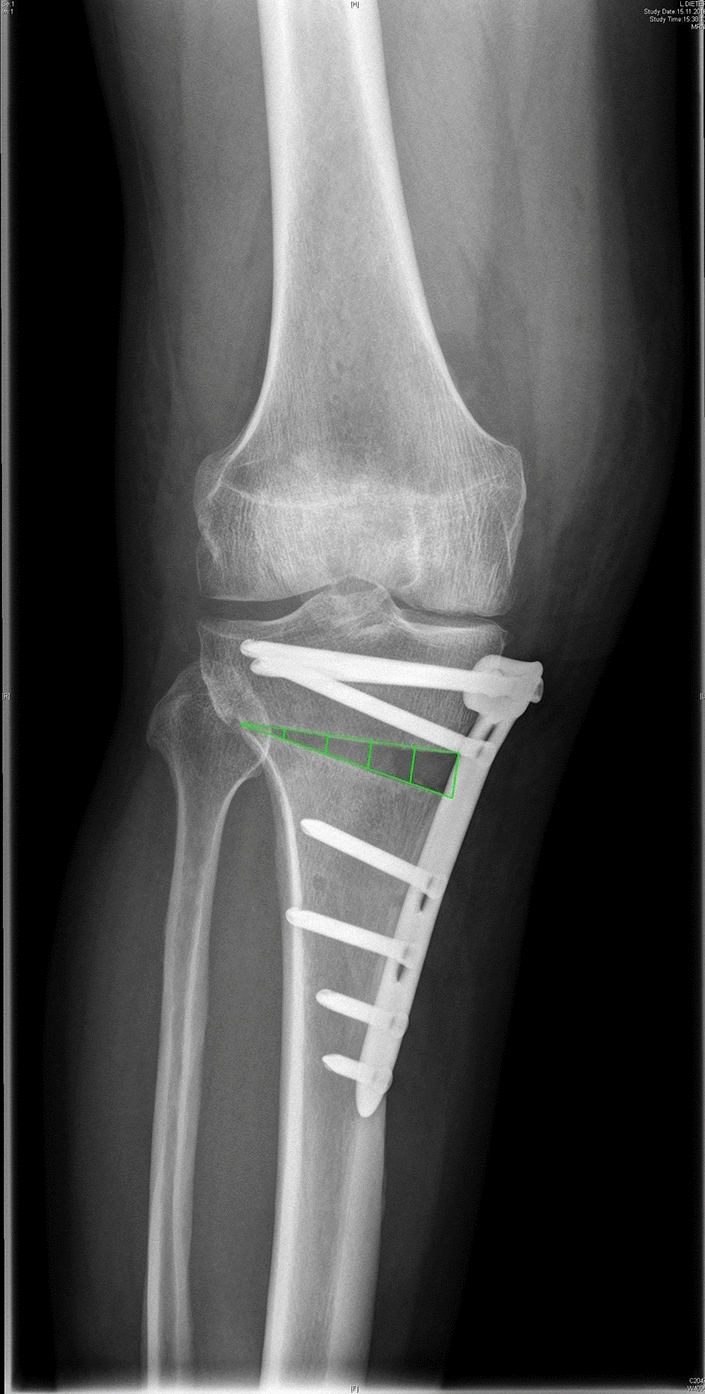
Radiographic Evaluation of an anteroposterior x-ray of a patients’ knee three days postoperatively. A triangle form of the osteotomy gap was divided into fifths by vertical lines, counting from the lateral to the medial cortex, to display the progress of consolidation. A fifth was rated as successfully consolidated when more than 50% of it was filled with bone

Axis correction was measured with the hip-knee-ankle (HKA) angle before and after surgery on a full-length x-ray of the lower limb in anterior–posterior view. Furthermore, at each postoperative appointment, anteroposterior as well as lateral x-rays of the knee were performed. Those anteroposterior x-ray images were evaluated according to the radiologic index of Brosset et al. [16] (see Fig. 1).

Specific consolidation aims were determined at the following appointments: 3 fifths filled after 6 weeks, 4 fifths with radiologic bone healing 12 weeks after surgery and all fifths should display consolidation 6 and 12 months postoperatively. Evaluation was performed by an experienced senior orthopedic and trauma consultant. A slope for each patients’ consolidation was created with a linear regression, using the slopes’ gradient as an indicator for the acceleration of consolidation [17]. GNU Image Manipulation Program (Spencer Kimball, Peter Mattis and GIMP development team) was used for analysis.

### Imaging evaluation

To control physiological bone healing and perfusion, CEUS and DCE-MRI examinations and evaluations of the osteotomy gap were implemented, following established protocols as used previously [10–12].

CEUS of the osteotomy gap was performed at every appointment. Therefore, we used established parameters: peak enhancement (PE in arbitrary units [a.u.]) as the maximum signal intensity of the enhancement curve, wash-in rate (WiR [a.u.]) as the steepest slope of the curve as well as wash-in perfusion index (WiPi [a.u.]) as the ratio between wash-in area under the curve and rise time [12]. CEUS examinations were assessed by the dedicated quantification software VueBox (Bracco Imaging, Milan, Italy).

DCE-MRI evaluated bone perfusion of the osteotomy gap 12 weeks postoperatively. Images of the DCE-MRI were postprocessed by a dedicated quantification software (Tissue4D, Siemens Healthcare, Erlangen, Germany), and the ratios between the initial area under the enhancement curve of the osteotomy gap relative to the muscle tissue were assessed.

**Fig. 2:**
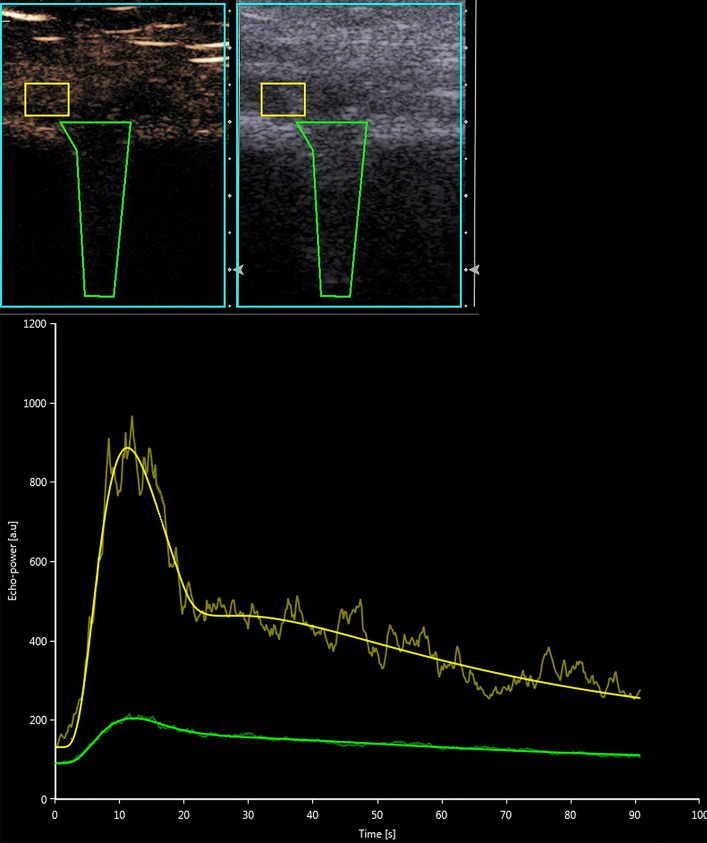
Contrast-enhanced ultrasound (CEUS) examination and evaluation of an osteotomy gap (patient without ABG). Upper part: sonography of the osteotomy gap with the specific contrast-enhanced cadence mode (left) and the standard b-mode (right). Lower part: evaluation over time of both regions of interests (ROI) with the dedicated quantification software VueBox (Bracco Imaging). Green: ROI 1 in the osteotomy gap. Yellow: ROI 2 in adjacent tissue without fascia or vessels (colour figure online)

**Fig. 3:**
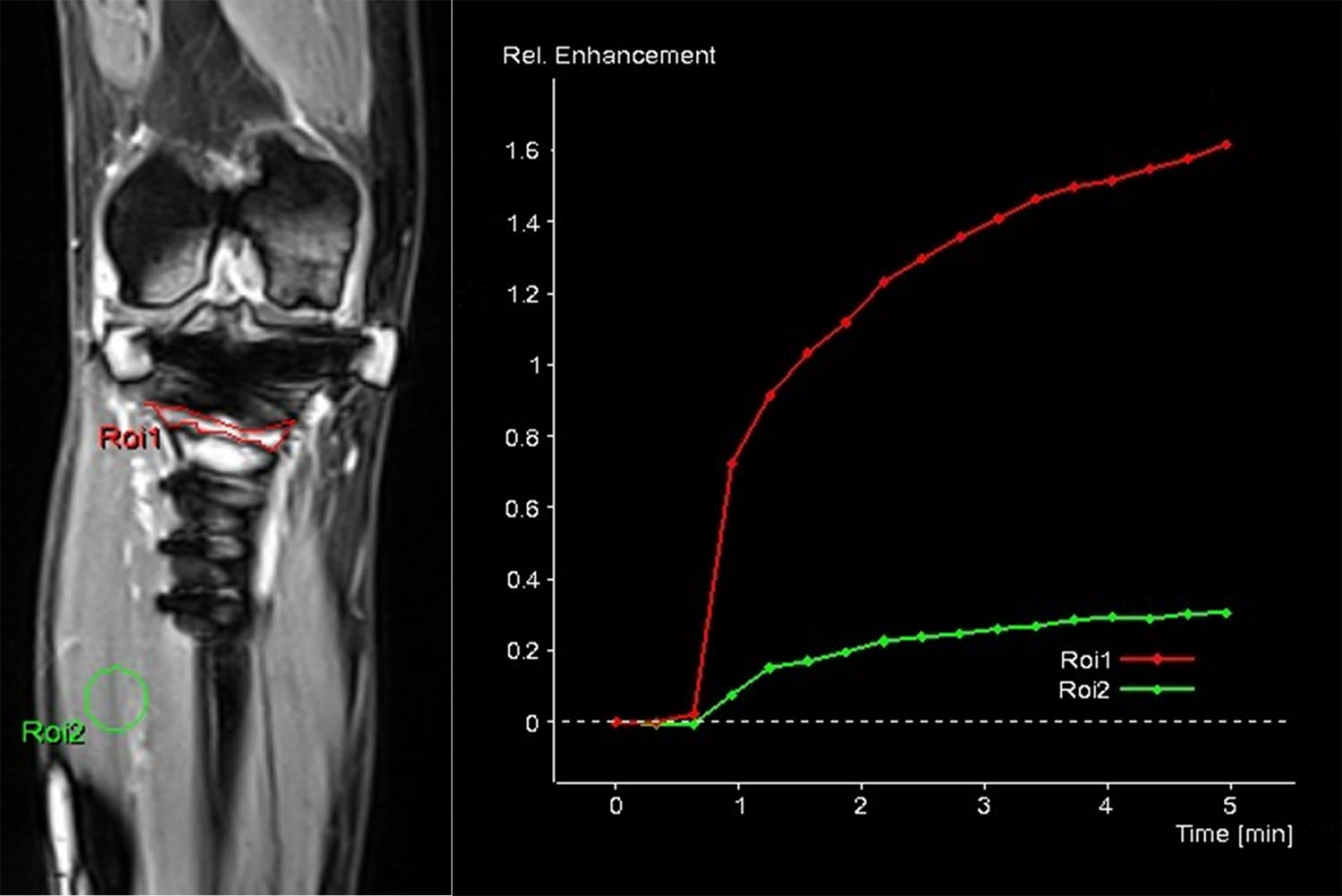
Dynamic magnetic resonance imaging (DCE-MRI) examination and evaluation of an osteotomy gap (patient without ABG). Left: DCE-MRI of the osteotomy gap. Right: evaluation over time of both regions of interests (ROI) with the dedicated quantification software Tissue4D (Siemens Healtcare). Red: ROI 1 in the osteotomy gap. Green: ROI 2 in adjacent muscle tissue (colour figure online)

An example of an examination and evaluation of CEUS is illustrated in Fig. 2 and of DCE-MRI in Fig. 3.

### Data analysis

Statistical analysis was performed using Stata statistical software (version 16.1, StataCorp, United States). Data were described by using means and standard deviations or medians and interquartile ranges (IQR), as appropriate. Microsoft® Excel® for Microsoft 365 MSO (16.0.14326.21008) was used to generate the graphs.

Because of the sample size of each group, we assumed data not to be normal distributed, so the Wilcoxon sign rank test was used to compare both groups. Categorical data were analyzed by using the Fisher exact test. A p-value < 0.05 was considered to be statistically significant.

A binomial logistic regression was performed to determine the effect of the gap size and age on the consolidation.

### A priori sample size calculation

An a priori sample size estimation was conducted. G*Power 3 [18] was used for all sample size estimations. Considering a difference of 1 fifths in consolidation with a standard deviation of 0.8 fifths between the two groups as a clinically relevant change, an effect size (Cohen’s d) of 1.25 can be assumed. To detect a significant difference between the mean of two independent groups, a sample size of 15 subjects per group is needed to achieve a power of > 90%. Recruitment was continued until at least 15 subjects in each group successfully finished one year of follow-up.

## Results

### Radiographic evaluation

Radiologically, 97.1% of all osteotomy gaps consolidated within the one-year follow-up. Only one patient without ABG developed a radiographic non-union without clinical symptoms. Radiologic evaluation showed a significantly better consolidation of the osteotomy gaps in patients with ABG 12 months after surgery: Only 60% of group A had all fifths filled with osseous tissue compared to 100% in group B (*p* = 0.006). Regarding the x-ray evaluation at 6 weeks and 6 months postoperatively, a tendency of faster bone healing with ABG was apparent but could not be proven with statistical significance (6 weeks (3 fifths filled): 50% in group A vs. 80% in group B, *p* = 0.089; 6 months (all fifths filled): 30% vs. 60%, *p* = 0.097) (Table 2). While patients without ABG had in general a slower consolidation in radiographic imaging, patients with graft transplantation experienced rapid progress of bone consolidation resulting in a steeper slope. Concerning the gradient of the consolidation slopes over time, a significantly increased bone healing of the osteotomy gaps with graft transplantation could be detected (*p* = 0.012).

**Table 2:**
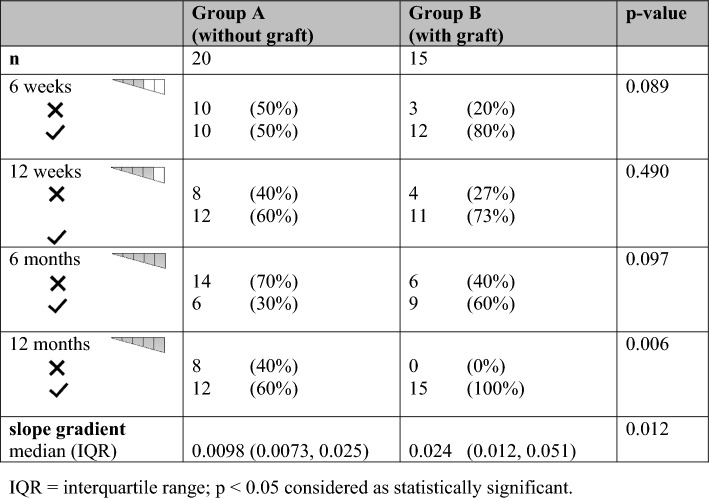
Radiographic results over time with the consolidation classification in fifths

The pre- and postoperatively measured HKA angles showed a tendency that patients with larger correction angles received ABG (6.0 vs. 8.0 degrees; *p* = 0.053).

Using the binominal logistic regression, we found a non-significant correlation between the gap size and those from Group A who did not reach the consolidation goal after one year with odds ratio (OR) = 0.733 and *p* = 0.070. After adjustment for age, the OR was 0.699 with *p* = 0.052.

### Imaging evaluation

CEUS and DCE-MRI did not show any significant differences regarding the perfusion of the osteotomy gaps between the two groups as well as physiological bone healing without indication of an infection or impaired perfusion (*p* > 0.05 for all parameters).

### Functional assessment

**Fig. 4:**
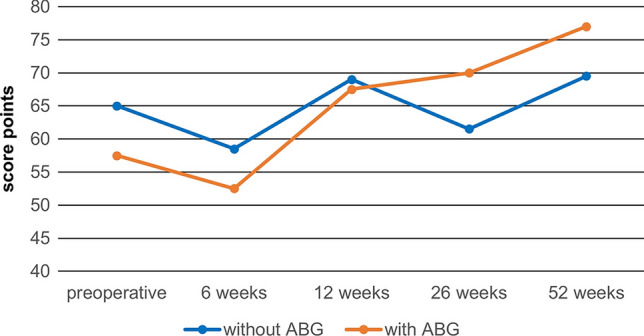
Medians over time of group A (without ABG) and group B (with ABG) of part 1 of the KSS. Part 1 of the KSS is the knee score and includes range of motion, stability, pain, alignment, flexion contractures and extension lag

There were no significant differences regarding the functional tests of patients with or without grafting, but there was a tendency of better function and less pain in patients receiving ABG (p = 0.290 after one year) (Fig. 4). Part 1 of the KSS is the knee score section and includes range of motion, stability, pain, alignment, flexion contractures and extension lag.

**Fig. 5:**
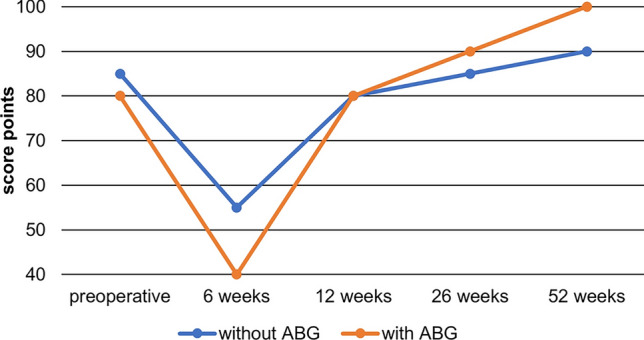
Medians over time of group A (without ABG) and group B (with ABG) of part 2 of the KSS. Part 2 is the functional score section and includes walking, stair climbing as well as the necessity and type of walking aids

Part 2 of the KSS is the functional score section and includes walking, stair climbing as well as the necessity and type of walking aids. In Part 2, patients receiving ABG also showed a tendency of better function without significant results (*p* = 0.170 after one year) (Fig. 5).

Concerning the LKS, group A scored a median of 65.5 points (IQR: 48.5–74) and group B of 43 points (IQR: 30–66) preoperatively (*p* = 0.071). One year after surgery, the median of group A was 72 points (IQR: 71–84) and the median of group B 89 points (IQR: 71–95) (*p* = 0.640).

In the mental part of the SF12, there were no significant differences between patients with or without graft transplantation before and one year after surgery (preoperative: *p* = 0.61; 1 year postoperative: *p* = 0.46).

Regarding the physical part of the SF12, group A scored preoperatively a median of 39.5 points (IQR: 29.0–46.2) and group B of 31.6 points (IQR: 26.9–38.8) (*p* = 0.170). At the one-year follow-up, group B reached slightly better results with a median of 48.4 points (IQR: 38.9–54.5) than group A with 43.8 points (IQR: 37.6–51.7) (*p* = 0.640).

## Discussion

The most important finding of the present study was that bone grafting resulted in an earlier consolidation of the osteotomy gap. Moreover, grafting seemed to increase the complete osseous union of the gap and showed a tendency to a better functional outcome after one year.

Among others, grafts were typically used to fill open wedge osteotomy gaps to stabilize the osteotomy and to secure bone consolidation [4]. Nowadays, it is controversially discussed when to perform grafting as there are several studies with good results without grafts [19–21].

Zorzi et al. [4] conducted a randomized, controlled study to investigate the advantages of using grafts. In this study with 46 patients, they discovered a complete consolidation after 12 weeks regardless of grafting. No statistical differences between the two groups according to the consolidation of the osteotomy gap were evident. However, no consolidation status over time was contained and no evaluation tools like the triangle tool by Brosset et al. [16] were used to assess consolidation semi-objectively. In line, a systematic review of Slevin et al. [7] found the same consolidation rates and no clinically significant advantage of using bone void fillers. Likewise, no chronological sequences of consolidation rates were evaluated. Similar, the meta-analysis of Han et al. [6] reported the same time for consolidation, union rate or complications independent of grafting. Furthermore, Silboni et al. [22] also did not recommend the use of grafts in OWHTO with locking plates or corrections smaller than 10° because they did not find any statistically significant differences between both groups.

In contrast to these studies, Fucentese et al. [8] observed in a prospective, randomized study an increased bone healing rate in patients with ABG 3 and 12 months after surgery. Performing a computed tomography, they found that 91.5% of the osseous gaps were healed at one-year follow-up in patients with graft compared to 59.1% in patients without bone void fillers. These results were confirmed by Ulucaköy et al. [23]. In their study with 25 patients, a faster healing of the osteotomy gap in patients with graft and a prolonged consolidation time in larger gaps were observed. This is in line with our data that also indicated an accelerated bone union in patients with bone transplantation, especially after one year (100% vs. 60%).

Furthermore, Pornrattanamaneewong et al. [24] assumed that a remaining medial gap of larger than 5 mm could lead to a higher risk for proximal tibia fractures when there is a need to remove the osteosynthesis. Our results show that a completely filled gap is more likely achieved with ABG and therefore might hold future benefits if a removal of the osteosynthesis is necessary, for example due to implant-related pain or implantation of a knee endoprosthesis.

Looking at the functional scores, Fucentese et al. [8] and Ulucaköy et al. [23] could not find significant differences between patients receiving grafts and patients without bone transplantation. Therefore, both did not recommend the routine use of grafts. We also detected no statistically significant differences in the functional scores, but we assume the data not to be statistically significant due to the limited number of patients. Nevertheless, we found a clear tendency toward better functional results with grafts. 6 weeks postoperative, patients with graft transplantation achieved lower functional scores, possibly caused by pain of the ipsilateral donor location. These patients with bone grafts had initially worse values in both parts of the KSS and in the LKS before surgery but ended up with higher values than patients without grafting one year later.

Additionally, ABG were used significantly more often in patients with nicotine abuse. According to the operation reports, the surgeons often decided intraoperatively to use bone grafting depending on their patients’ smoking status. Although smoking has an inhibiting effect on bone healing and can lead to delayed bone union, non-union [25, 26] or to a subjective worse outcome [27], the osteotomy gaps consolidated significantly faster with the use of bone grafts. These patients also had better functional outcomes compared to the group without ABG and less smokers. Aryee et al. [28] also suggested to use ABG in patients with heavy smoking or other risk factors like obesity or a large opening gap with more than 10 mm. Silboni et al. [22] agreed with those recommendations without regard to nicotine abuse but to lateral cortex hinge fractures.

In this study, CEUS and DCE-MRI were used as control examinations of physiological osseous healing and did not show any significant differences regarding graft transplantation. Compared with the results of previous studies published by Fischer et al. [9–11], a normal perfusion for bone healing processes could be detected. Accordingly, the endpoint of bone healing could be investigated specifically regarding ABG while controlling for other potentially confounding risk factors such as infection or impaired perfusion.

Some findings of this study did not reach statistical significance, which may be due to the small sample size and different donor locations. Furthermore, these results cannot be generalized for a larger population due to the limited number of patients in our trial. More well-powered randomized controlled trials are needed to differentiate the impact of the donor site on functional results. Another limitation of this study is the evaluation of consolidation only by x-ray. Although the radiographic assessment was highly standardized, the risk of the appearance of the osteotomy gap, the bone graft or the position of the ABG in the osteotomy gap influencing the corresponding results cannot be fully excluded. For a more accurate evaluation of consolidation, a computed tomography would be necessary, but for reasons of radiation protection cannot be performed four times within a year for research purposes. Thus, CEUS and DCE-MRI were performed to gather more indications for a physiological bone healing process.

In conclusion, we assume that autologous bone grafting induces earlier osseous consolidation after medial OWHTO. We see a tendency toward better functional results without reaching significance. ABG was used significantly more often in patients with nicotine abuse in this study and showed good 1-year outcomes. We hypothesize that these high-risk patients may benefit from complete and timely consolidation. Thus the use of ABG may be useful in those cases.
